# Development of an Enzymatic Biosensor Using Glutamate Oxidase on Organic–Inorganic-Structured, Electrospun Nanofiber-Modified Electrodes for Monosodium Glutamate Detection

**DOI:** 10.3390/bios13040430

**Published:** 2023-03-28

**Authors:** Hamdiye Atilgan, Betul Unal, Esra Evrim Yalcinkaya, Gizem Evren, Gozde Atik, Fatma Ozturk Kirbay, Nur Melis Kilic, Dilek Odaci

**Affiliations:** 1 Department of Biochemistry, Faculty of Science, Ege University, Bornova, Izmir 35100, Turkey; 2 Department of Chemistry, Faculty of Science, Ege University, Bornova, Izmir 35100, Turkey

**Keywords:** nanobiotechnology, electrochemical method, nanomaterial, nanofiber, MSG, montmorillonite

## Abstract

Herein, dendrimer-modified montmorillonite (Mt)-decorated poly-Ɛ-caprolactone (PCL) and chitosan (CHIT)-based nanofibers were prepared. Mt was modified with a poly(amidoamine) generation 1 (PAMAM_G1_) dendrimer, and the obtained PAMAM_G1_–Mt was incorporated into the PCL–CHIT nanofiber’s structure. The PCL–CHIT/PAMAM_G1_–Mt nanofibers were conjugated with glutamate oxidase (GluO*x*) to design a bio-based detection system for monosodium glutamate (MSG). PAMAM_G1_–Mt was added to the PCL–CHIT backbone to provide a multipoint binding side to immobilize GluO*x* via covalent bonds. After the characterization of PCL–CHIT/PAMAM_G1_–Mt/GluO*x*, it was calibrated for MSG. The linear ranges were determined from 0.025 to 0.25 mM MSG using PCL–CHIT/Mt/GluO*x* and from 0.0025 to 0.175 mM MSG using PCL–CHIT/PAMAMG_1_–Mt/GluO*x* (with a detection limit of 7.019 µM for PCL–CHIT/Mt/GluO*x* and 1.045 µM for PCL–CHIT/PAMAMG_1_–Mt/GluO*x*). Finally, PCL–CHIT/PAMAMG_1_–Mt/GluO*x* was applied to analyze MSG content in tomato soup without interfering with the sample matrix, giving a recovery percentage of 103.125%. Hence, the nanofiber modification with dendrimer-intercalated Mt and GluO*x* conjugation onto the formed nanocomposite structures was performed, and the PCL–CHIT/PAMAM_G1_–Mt/GluO*x* system was successfully developed for MSG detection.

## 1. Introduction

Glutamate (Glu) is an amino acid in protein-containing foods [1]. Numerous studies have discovered that Glu is present in the cerebral cortex in one of the major intracellular signal pathways. Changes in the Glu concentration cause Huntington’s disease [2,3]. Moreover, Glu is a crucial indicator for various other illnesses [4], such as musculoskeletal pain [5], tumors [6], and Alzheimer’s disease [7]. Currently, Glu has been detected using multiple neurochemical probes, including carbon fiber microsensors based on enzymes or microdialysis [8]. Enzymatic biosensors utilize glutamate oxidase (GluO*x*) and glutamate dehydrogenase (GDH) as recognition components to detect Glu. Monosodium glutamate (MSG), a form of Glu, is a commonly used food additive that increases food’s palatability [9], and it is hazardous. MSG releases neurotransmitters crucial to healthy physiological and pathological processes by acting on Glu receptors [10]. Excessive ingestion of MSG can cause health problems such as headaches, stomachaches, and neuronal excitotoxicity [11]. Thus, detecting MSG content in food is important to identify whether its amount exceeds permissible limits [12]. Therefore, developing reliable, fast, and specific methods for MSG detection is critical. Numerous electrochemical MSG biosensors have been created so far. For example, Devi et al. developed a novel immunosensor using gold nanoparticles decorated on a molybdenum disulfide/chitosan matrix for MSG detection [13]. In another study, Sharma et al. fabricated an immunosensor using a gold–chitosan nanocomposite to immobilize the antibody against MSG [14]. Moreover, a GluO*x*-based hybrid nanoflower and horseradish peroxidase were successfully prepared to design an MSG biosensor [15].

Target molecules may be identified with great sensitivity and selectivity using electrochemical methods, such as amperometry, impedimetry, and potentiometry [16,17]. Due to their mobility, sensitivity, simplicity, and ease of miniaturization and integration [18], electrochemical sensors are some of the most promising means for in vivo and onsite monitoring of biomolecules [19,20]. The most important point in preparing electrodes is the selection of materials while modifying electrodes with biological molecules [21]. Various materials, such as polymers, nanomaterials, and clays, can be selected to cover the electrode surface. Clays are inorganic materials, which are preferred to design biosensors because of their high adsorption capacity and stability [22]. Amino acids, calixarenes, and other numerous organic materials have been used to intercalate clays for their use in the design of biosensors [23,24,25,26,27,28]. Poly(amidoamine) (PAMAM) dendrimers with different generations were previously used to modify montmorillonite (Mt) clays [29,30]. PAMAM has also been used to create dendrimer-modified enzyme biosensors [31], DNA biosensors [32], immunosensors [33], and chemical sensors [34]. Nanomaterials with large surface areas and carefully spaced functional sites on their surfaces have been suggested to increase sensitivity, target molecule accessibility, provide quicker mass transfer rates, and shorten biosensor reaction times [35]. Electrospun nanofibers (ESNFs) can be considered suitable for supporting the immobilization of biorecognition elements because they meet several requirements, including maximal contact with the surrounding media, an extensively large surface area, a very porous structure, excellent surface modification, and subcellular size [36,37,38]. For this purpose, Chokkiah et al. synthesized polyvinyl alcohol (PVA)–polyaniline–graphitic carbon nitride ESNFs for chloride ion sensing to help environmental monitoring [39]. Yezer and Demirkol created cellulose acetate–chitosan/glucose oxidase ESNFs for sensing glucose [40]. Owing to its unique properties, chitosan (CHIT) has received attention for synthesizing ESNFs, among other carbohydrates [41]. Dendrimers have also been recently applied in bulk and on the surface to produce ESNFs in various ways [42].

Herein, PAMAM dendrimer-modified montmorillonite (Mt)-decorated poly-Ɛ-caprolactone (PCL) and chitosan (CHIT) electrospun nanofibers (PCL–CHIT/PAMAM_G1_–Mt ESNFs) were formed via electrospinning. CHIT was supplemented into the composition of PCL nanofibers because PCL is highly hydrophobic. PAMAM_G1_ with eight primary amino groups was used to modify Mt. The presence of Mt provides mechanical stability for the matrix immobilization to supply the multipoint attachment of the enzyme. To fabricate PCL–CHIT/PAMAM_G1_–Mt ESNFs without beads and flat, various solvent systems were examined to find the best combination for electrospinning application. Then, PCL–CHIT/PAMAM_G1_–Mt was characterized using scanning electron microscopy (SEM). Afterward, GluO*x* was immobilized onto PCL–CHIT/PAMAM_G1_–Mt ESNFs. Cyclic voltammetry (CV) and differential pulse voltammetry (DPV) were used to prove the success of immobilization. The designed PCL–CHIT/PAMAM_G1_–Mt/GluO*x* was facile and had a reduced detection threshold with the largest detection range. In addition, this method offers a technique for monitoring MSG levels in food and beverages.

## 2. Materials and Methods

### 2.1. Materials

PAMAM dendrimer generation 1.0 solution (PAMAM_G1_; [NH_2_(CH_2_)_2_NH_2_]:(G = 1); dendriPAMAM(NH_2_)8; Mw = 1429.85 g/mol), glutamate oxidase (GluO*x*; L-glutamate: oxygen oxidoreductase, EC 1.4.3.11, from *Coriolus* sp., 0.2 U/vial), L-glutamic acid monosodium salt monohydrate (MSG; MW = 187.13 g/mol), polycaprolactone (6-Caprolactone polymer; average Mn = 80,000), glutaraldehyde (25%), formic acid (98–100%), D-glucose (99.5%), ascorbic acid (99%), 3-acetamidophenol (97%), uric acid (99%), L-glycine, L-lysine, L-aspartic acid, potassium hexacyanoferrate (K_3_[Fe(CN)_6_]), monosodium phosphate (NaH_2_PO_4_), and potassium chloride (KCl) were purchased from Sigma-Aldrich, St. Louis, MO, USA. Chitosan middle-viscous was purchased from Fluka, Buchs, Switzerland and acetone was provided by Merck, Darmstadt, Germany. The water was used from a Millipore (Burlington, MA, USA) Milli-Q ultrapure water system. Powder tomato soup was purchased from local market to detect MSG in real samples.

### 2.2. Instrumentation

The characterizations of neat Mt and PAMAM_G1_-modified clay (PAMAM_G1_–Mt) were performed via Fourier transform infrared (FTIR) spectroscopy, X-ray diffraction (XRD), and thermogravimetric analysis–differential thermogravimetry (TGA–DTG) methods. The structural characterization of the samples was first performed with FTIR analysis using the PerkinElmer Spectrum 100 FTIR Spectrometer. For this purpose, the samples were mixed well with potassium bromide (KBr), and fine pellets were prepared. The FTIR of the pellets was performed in the range of 4000–500 cm^−1^. In addition, neat Mt and PAMAM_G1_–Mt were characterized using XRD (Panalytical X’Pert Pro, Malvern, UK; Cu-Kα, λ = 1.54056 Å). The *d*-values (001) of interlayer distances can be calculated based on diffraction angles (2*θ*) obtained via the XRD method using Bragg’s law (*n λ* = 2 *d* sin *θ*). Here, *d* is the distance between the layers, *θ* is the diffraction angle, *n* is the number of waves, and *λ* is the waveform of the transmitted light. Thermal degradation of the samples was examined using TGA–DTG (Perkin Elmer Pyris 1 TGA–DTG, Waltham, MA, USA). They were decomposed under nitrogen gas at 10 °C/min with increments from room temperature to 800 °C. The main purpose of TGA is to determine the weight loss and the degradation temperatures of the neat clay and modified clay mineral.

PCL–CHIT nanofibers were collected using NanoWeb Electrospin 103 (MaviTech, Istanbul, Turkey) onto indium tin oxide surfaces (ITO; 24 mm × 24 mm; resistance = 8–10 Ω/sq, thickness = 150–170 µm; from Teknoma Inc., Izmir, Turkey), and the contact angles of these ESNF-covered ITO surfaces were measured using an Attension Theta goniometer. The morphological structures of PCL–CHIT, PCL–CHIT/Mt, PCL–CHIT/PAMAM_G1_–Mt, and PCL–CHIT/PAMAM_G1_–Mt/GluO*x* were characterized using SEM (Zeiss Sigma 300) and SEM–energy dispersive spectrometry (EDS). Amperometry, CV, and DPV measurements were performed using a PalmSens potentiostat (PalmSens Instruments, Houten, The Netherlands) at room temperature. Electrochemical impedance spectroscopy (EIS) analysis utilized a CHI 6005 C electrochemical analyzer (CH Instruments Incorporated, Austin, TX, USA). A triple electrode system, including a glassy carbon electrode (GCE), a platinum electrode (BASI, West Lafayette, IN, USA, and an Ag/AgCl reference electrode (Metrohm, Herisau, Switzerland), was used for all electrochemical measurements. CV, DPV, and EIS measurements were realized in 50 mM sodium phosphate buffer at pH 6.5 containing 5.0 mM K_3_[Fe(CN)_6_] and 0.1 M KCl.

### 2.3. Modification of Mt with PAMAM_G1_ Dendrimer

Montmorillonite (Mt) was modified with PAMAM_G1_ via the cation exchange process. During this process, Na^+^ ions between the Mt interlayers were exchanged with the quaternary alkyl ammonium ions in the PAMAM_G1_. For this purpose, 0.5 g of Mt clay mineral was dispersed in 200 mL deionized water at room temperature overnight [29,30]. Simultaneously, 50 mL of a 0.02 mmol PAMAM_G1_ solution and an equivalent amount in relation to the cation exchange capacity of Mt was prepared in a different beaker and stirred. An aqueous, 1.0 M HCl solution was added to adjust the pH to 2.0–3.0 [29,30]. After stirring for a few hours, the protonated PAMAM_G1_ solution was slowly added to the neat Mt dispersion and left at room temperature overnight. The obtained PAMAM_G1_-modified clay (PAMAM_G1_–Mt) was precipitated using ultracentrifugation (at 18,000 rpm for 15 min). PAMAM_G1_–Mt was washed with distilled water at least three times and filtered until no bromide ions were detected using an aqueous silver nitrate (AgNO_3_) solution [29,30]. The resulting sample was dried in a vacuum at 35 °C.

### 2.4. Preparation of the PCL–CHIT/PAMAM_G1_–Mt/GluOx Biosensors

A 10% (*w*/*v*) polycaprolactone (PCL) solution and a 0.5% (*w*/*v*) chitosan solution were separately prepared from 3:7 (*v*/*v*) formic acid to acetone solutions. PCL and CHIT solutions were mixed in a ratio of 7:3 (*v*/*v*), then stirred overnight at room temperature [29,30]. The solutions were filled into 2.0-mL syringes fitted with an 8.8 mm inner diameter metallic needle. The distance between the clean GCE, which was attached to the collector plate, and the syringe, which was fixed horizontally in a syringe pump (NE-300; New Era Pump Systems, Inc., New York, NY, USA) (tip-to-collector distance), was 18–19 cm in the electrospinning unit. The applied voltage and flow rate of the polymer solution were adjusted to ~20 kV and ~1.28 mL/h, respectively. After the electrode was covered with PCL–CHIT nanofibers, it was dried at 40 °C for 1 h. Then, 0.5% (*w*/*v*) Mt or PAMAM_G1_–Mt was first added to a 7:3 (*v*/*v*) PCL–CHIT solution to prepare PCL–CHIT/Mt and PCL–CHIT/PAMAM_G1_–Mt solutions, respectively [29,30]. The homogeneous solution was mixed overnight at room temperature and then poured into the syringes. The distance between the clean GCE and the syringe was 17–19 cm in the electrospinning unit. The applied voltage and flow rate of the polymer solution were ~19 kV and ~0.6 mL/h, respectively. Electrodes coated with bead-free PCL–CHIT/Mt and PCL–CHIT/PAMAM_G1_–Mt nanofibers were dried at 40 °C for 1 h. Then, 10 µL of GluO*x* (0.2 U) enzyme was immobilized onto these electrodes using 2.5 µL glutaraldehyde (GA, 1.5%) as a cross-linking agent [17,18,31,40,43]. Afterward, the electrode was dried in an oven at 25 °C for 15 min. These modified PCL–CHIT/Mt/GluO*x* and PCL–CHIT/PAMAM_G1_–Mt/GluO*x* were utilized in electrochemical measurements.

### 2.5. Electrochemical Measurements

All amperometric measurements were performed in a 10 mL electrochemical working medium containing 50 mM, pH 6.5 sodium phosphate buffer at −0.7 V versus an Ag/AgCl electrode at room temperature, and responses were recorded in µA. For the surface characterization of bare GCE, GCE/PCL–CHIT/PAMAM_G1_–Mt, and GCE/PCL–CHIT/PAMAM_G1_–Mt/GluO*x*, CV, DPV, and EIS measurements were carried out in 50 mM, pH 6.5 sodium phosphate buffer containing 5.0 mM potassium hexacyanoferrate (K_3_[Fe(CN)_6_]) and 0.1 M KCl buffer. The CV and DPV measurements of bare GCE, PCL–CHIT/PAMAM_G1_–Mt, and PCL–CHIT/PAMAM_G1_–Mt/GluO*x*-coated GCE were conducted at potential rates of −0.4 to +0.8 V and −0.1 to +0.5 V with the scan rates of 50 and 25 mV/s, respectively. The EIS measurements of these GCEs were performed with frequencies in the range of 0.21 × 10^−4^ to 10 kHz and an excitation voltage of 0.18 V, and superimposed on a dc potential of 0.01 V in the same electrochemical working medium.

## 3. Results

### 3.1. Characterization of PAMAM_G1_–Mt

As a 2:1 layered silicate, montmorillonite minerals consist of an octahedral layer between two tetrahedral layers. There are exchangeable cations between the Mt interlayers, which can easily be replaced by other cations, such as quaternary ammonium cations coming into the clay’s environment via the cation exchange reactions. This method is performed to make hydrophilic silicate surfaces organophilic and to increase the layer spacing of the clay mineral [29,30]. The surface of Mt needs to be more hydrophobic to obtain the nanoscale dispersed Mt layers within the polymer matrix. For this purpose, Mt was modified with PAMAM_G1,_ which contained quaternary alkyl ammonium salt, and was characterized using the FTIR, XRD, and TGA–DTG methods.

**Figure 1:**
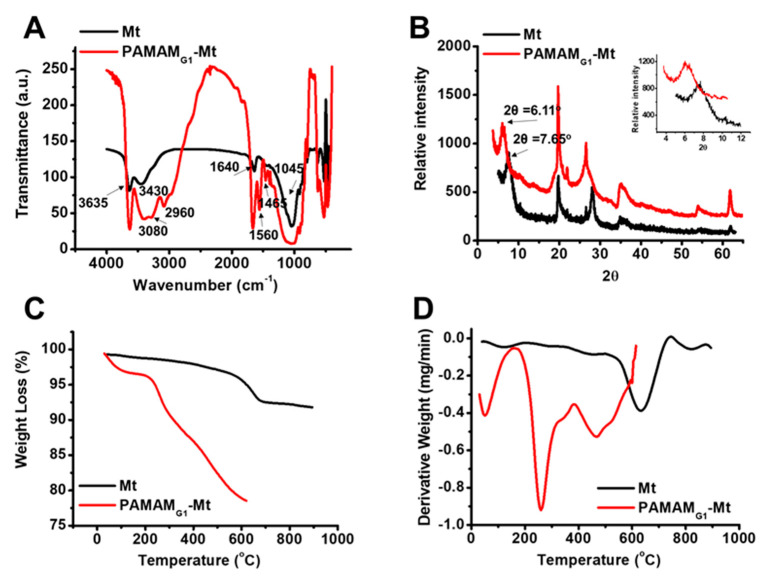
(**A**) Fourier transform infrared (FTIR) spectrum, (**B**) X-ray diffraction (XRD) pattern, (**C**) thermogravimetric (TG) analysis, and (**D**) differential thermogravimetry (DTG) thermograms of Mt and PAMAM_G1_-Mt.

Firstly, the structural characterization of Mt and PAMAM_G1_–Mt was performed using FTIR (Figure 1A). From the FTIR spectrum of pure Mt, the band that appeared at ~3635 cm^−1^ is attributed to the relative humidity in the Mt clay structure. The characteristic O–H stretching vibration of water between the layers appeared at ~3430 cm^−1^. Another band was detected at 1640 cm^−1^ owing to the O–H bending vibrations of hydrated water molecules. Additionally, a strong band observed at ~1045 cm^−1^ is attributed to the Si–O–Si and Si–O–Al stretches. After modification with PAMAM, the characteristic PAMAM_G1_ bands were also observed in the FTIR spectrum with Mt bands. The new bands were observed at 3080 and 2960 cm^−1^ owing to the –CH stretching vibrations in the PAMAM_G1_ structure. The strong band of –CH_2_ bending vibrations is assigned to 1465 cm^−1^; the bands of PAMAM_G1_ N–H amine groups are designated to ~1560 cm^−1^.

The changes in the basal spacing (d001) between the layers after exchanging sodium ions with the PAMAM_G1_ were detected using XRD analysis. The XRD patterns of Mt and PAMAM_G1_–Mt are presented in Figure 1B. The 2*θ* angle of Mt between layers was determined as 2*θ* = 7.65°, indicating the regular repeats of silicate layers in the range of 3°–9°. The basal spacing (d001) of Mt was calculated as 11.54 Å using the Bragg equation corresponding to the diffraction angle. After the intercalation of PAMAM_G1_ with Mt, the diffraction angle shifted to the lower values of 2*θ* = 6.11° in the same range. The basal spacing of PAMAM_G1_–Mt was expanded to 11.54 Å from 14.45 Å after modifying Mt with PAMAM_G1_. The increase in the basal spacing values indicates that the PAMAM_G1_ intercalates into the interlayer space of Mt. The interlayer distance increases with the alkyl chain length of the organic molecules. This result is comparable with our previous study on PAMAM with a variable alkyl chain length [29,30]. The basal spacing of the interlayers increases with the chain length of the PAMAM.

The thermal stability of Mt and PAMAM_G1_–Mt is presented with TG/–DTG curves in Figure 1C,D. From the TGA thermogram, Mt showed ~7.5% weight loss at 600 °C owing to the presence of volatile substances. In the DTG analysis of pure Mt, the degradation consists of two stages. The first weight loss was owing to the water adsorbed on the surfaces of the sheets at 0–120 °C. The second one arose from the loss of the adsorbed water in the inner parts of the layers owing to the dehydroxylation of the aluminosilicate lattices of the Mt structure at 635 °C [29]. The degradation of PAMAM_G1_–Mt occurred in three steps. In addition to these peaks, the degradation of PAMAM on the surface and between the Mt layers was observed at ~260 °C. After the modification, the degradation peak of Mt shifted toward the lower temperature (from 635 °C to 470 °C) compatible with the literature [44]. The amount of the organic cation content was determined to be 22.50% from the TG thermogram of the PAMAM_G1_–Mt, owing to the adsorption of PAMAM on the surface and between the clay layers.

### 3.2. Formation of PCL–CHIT/PAMAM_G1_–Mt and GluOx Conjugation on ESNFs

**Figure 2:**
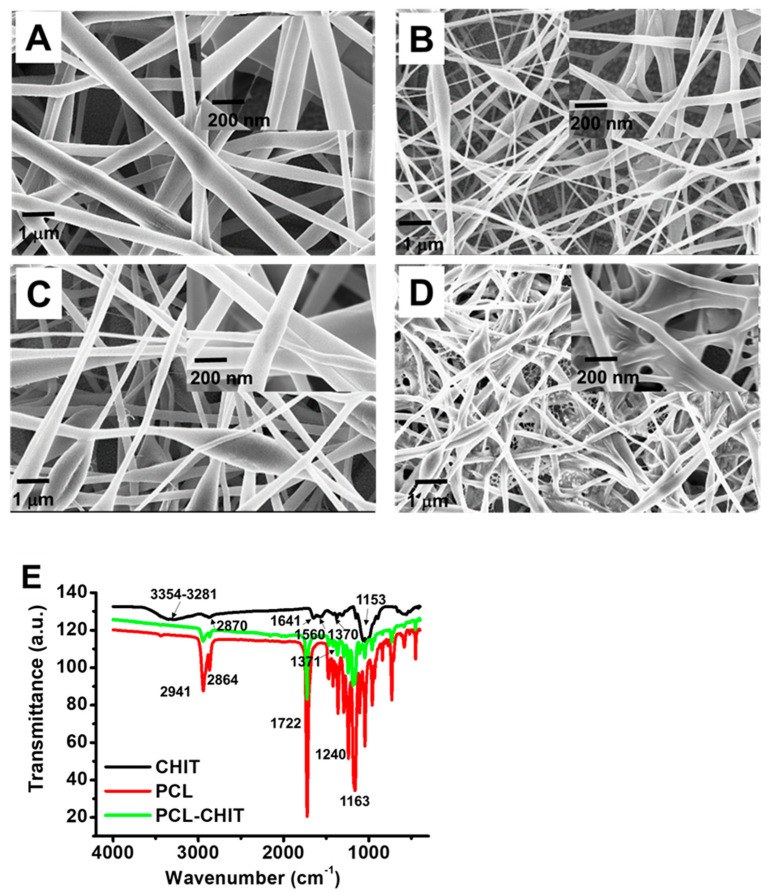
SEM images of (**A**) PCL–CHIT, (**B**) PCL–CHIT/Mt, (**C**) PCL–CHIT/PAMAM_G1_–Mt, and (**D**) PCL–CHIT/PAMAM_G1_–Mt/GluO*x* (insets show the higher-magnification SEM images). (**E**) FTIR spectra of PCL, CHIT, and PCL–CHIT ENFs.

In the SEM images of the PCL–CHIT nanofibers grown with Mt and PAMAM_G1_ (Figure 2A–C), the incorporation of clays decreased the diameter of the ESNFs. Figure 2D shows the SEM images of PCL–CHIT/PAMAM_G1_–Mt/GluO*x*. After the conjugation of GluO*x* on the PCL–CHIT/PAMAM_G1_–Mt, the morphology of nanofibers changed to a sticky form because glutaraldehyde created covalent bonds between the amine groups of GluO*x* and PCL–CHIT/PAMAM_G1_–Mt ESNFs. The structural characterization of PCL–CHIT nanofiber was performed using FTIR analysis (Figure 2E). Furthermore, the neat PCL and CHIT molecules were characterized via FTIR for their comparison with the nanofiber. According to the FTIR spectrum of neat CHIT, a strong, broad band in the region of 3354–3281 cm^−1^ corresponds to O–H and N–H stretching, namely, the intramolecular hydrogen bonds. The absorption band observed at ~2870 cm^−1^ can be attributed to C–H asymmetric stretching. These bands are the characteristics of polysaccharide molecules. The bands observed at ~1641 and 1560 cm^−1^ are attributed to C=O stretching and N–H bending, respectively. The C–H and O–H bending were confirmed using the bands that appeared at ~1414 and 1370 cm^−1^, respectively. The absorption band located at 1153 cm^−1^ relates to the asymmetric stretching of the C–O–C bridge. The bands observed at 1062 and 1026 cm^−1^ correspond to C–O stretching. All FTIR spectral bands of chitosan correspond with those in the literature [45,46]. The FTIR spectrum of neat PCL was also investigated, and the PCL absorption bands located at ~2941 and 2864 cm^−1^ were assigned to asymmetric and symmetric –CH_2_– stretching, respectively. The band observed at 1722 cm^−1^ represents the stretching vibration of the carbonyl group in PCL. Symmetric and asymmetric C–O–C stretching were observed as strong bands at ~1240 and 1163 cm^−1^ [47]. The similarity of the FTIR spectrum of the PCL–CHIT nanofiber with that of neat PCL is remarkable. Most CHIT bands coincided with neat PCL bands. Therefore, all CHIT bands were not observed, owing to the considerably lower percentage of chitosan and interference with the PCL bands. The band observed at ~1371 cm^−1^ (O–H bending) proves the existence of CHIT in the nanofiber.

**Figure 3:**
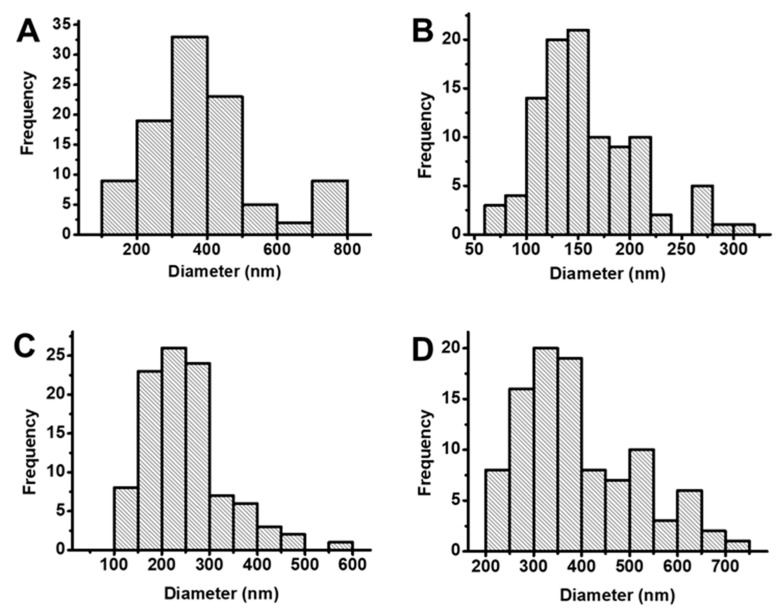
Histograms for diameter distributions of (**A**) PCL–CHIT, (**B**) PCL–CHIT/Mt, (**C**) PCL–CHIT/PAMAM_G1_–Mt, and (**D**) PCL–CHIT/PAMAM_G1_–Mt/GluO*x*.

SEM images were marked (100 different points) to evaluate the distributions of the nanofiber diameters using ImageJ. Figure 3 shows the histograms of the diameter distributions of PCL–CHIT, PCL–CHIT/Mt, PCL–CHIT/PAMAM_G1_–Mt, and PCL–CHIT/PAMAM_G1_–Mt/GluO*x*. The calculated diameter distributions of PCL–CHIT, PCL–CHIT/Mt, PCL–CHIT/PAMAM_G1_–Mt, and PCL–CHIT/PAMAM_G1_–Mt/GluO*x* were found as 356.61 ± 12.89, 141.33 ± 4.41, 227.15 ± 5.55, and 332.26 ± 10.73 nm, respectively. The addition of Mt to PCL–CHIT nanofibers decreased their size. As clays contain cations, they increase the conductivity of the solution [48]. The intercalation of Mt with PAMAM_G1_ and the decoration of PCL–CHIT nanofibers with PAMAM_G1_–Mt increased the nanofiber’s diameter. There was a decrease in conductance with the addition of organoclay, so the diameter of PCL–CHIT/PAMAM_G1_–Mt nanofibers increased [49]. Due to the swelling properties of CHIT and Mt, the diameters of PCL–CHIT/PAMAM_G1_–Mt nanofibers increase after GluOx immobilization in an aqueous solution [50,51].

**Figure 4:**
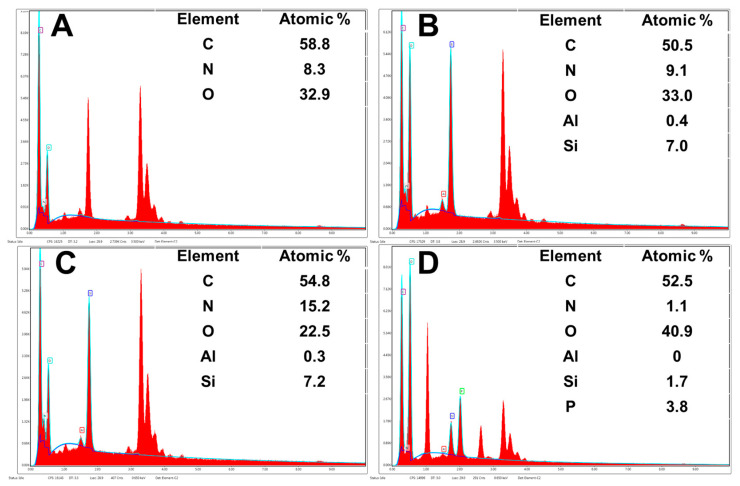
SEM–EDS results of (**A**) PCL–CHIT, (**B**) PCL–CHIT/Mt, (**C**) PCL–CHIT/PAMAM_G1_–Mt, and (**D**) PCL–CHIT/PAMAM_G1_–Mt/GluO*x*.

PCL is a biodegradable polyester with the chemical formula (C_6_H_10_O_2_)*_n_*, and chitosan (C_6_H_11_NO_4_)*_n_* is a copolymer of N-acetyl-D-glucosamine and D-glucosamine. According to the SEM–EDS results of the PCL–CHIT nanofibers, carbon, oxygen, and nitrogen elements were observed (Figure 4A). By adding Mt (Al_2_H_2_O_12_Si_4_) to the polymer solution, aluminum and silicon elements were seen in the PCL–CHIT/Mt nanofibers (Figure 4B). After the modification of Mt with the PAMAM_G1_ dendrimer, the percentage of nitrogen in PCL–CHIT/PAMAM_G1_–Mt also increased with the nitrogen in the PAMAM_G1_ structure ([NH_2_(CH_2_)_2_NH_2_]:(G = 1); dendri PAMAM(NH_2_)_8_) (Figure 4C). GluO*x* contains a riboflavin nucleic acid derivative (flavin adenine dinucleotide (FAD)) because of its flavoprotein structure. Nucleotides are bound via phosphodiester bonds in FAD [40]. Therefore, phosphorus was observed in PCL–CHIT/PAMAM_G1_–Mt/GluO*x* ESNFs after GluO*x* immobilization (Figure 4D). This indicated immobilization was successfully performed.

**Figure 5:**
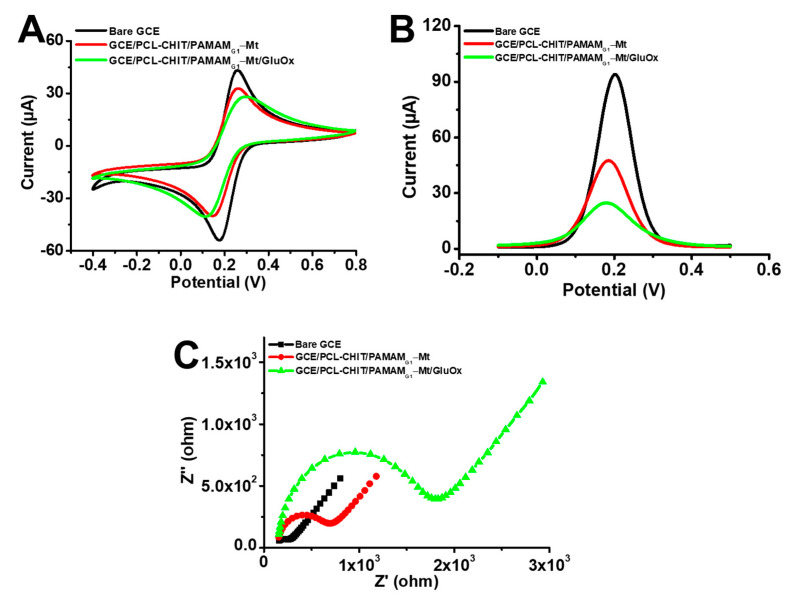
(**A**) Cyclic voltammograms of bare GCE, GCE/PCL–CHIT/PAMAM_G1_–Mt, and GCE/PCL–CHIT/PAMAM_G1_–Mt/GluO*x* at a scan rate of 50 mV.s^−1^. (**B**) Differential pulse voltammograms of bare GCE, GCE/PCL–CHIT/PAMAM_G1_–Mt, and GCE/PCL–CHIT/PAMAM_G1_–Mt/GluO*x* at a scan rate of 25 mV.s^−1^. (**C**) Nyquist plots for EIS of bare GCE, GCE/PCL–CHIT/PAMAM_G1_–Mt, and GCE/PCL–CHIT/PAMAM_G1_–Mt/GluO*x* at +0.18 V (all measurement cells included 5.0 mM K_3_[Fe(CN)_6_] and 0.1 M KCl in a pH 6.5, 50 mM phosphate buffer).

The electrochemical surface of the developed biosensor was characterized using CV, DPV, and EIS. K_3_[Fe(CN)_6_] was used as a redox probe during electrochemical measurements. According to cyclic voltammograms, peak currents were calculated as 43.194, 31.831, and 28.113 µA for bare, PCL–CHIT/PAMAM_G1_–Mt, and PCL–CHIT/PAMAM_G1_–Mt/GluO*x*-modified GCEs, respectively. Redox peak potential separations were 0.082, 0.116, and 0.186 mV for bare, PCL–CHIT/PAMAM_G1_–Mt, and PCL–CHIT/PAMAM_G1_–Mt/GluO*x*-modified GCEs, respectively. As shown in Figure 5A, the current responses decreased after each modification owing to the limitation of the K_3_[Fe(CN)_6_] transfer to the electrode surface. As the CV results, the peak currents gradually decreased as the electrode surfaces were covered with ESNFs. They were calculated as 92.750, 46.108, and 21.864 µA for bare, PCL–CHIT/PAMAM_G1_–Mt, and PCL–CHIT/PAMAM_G1_–Mt/GluO*x*-modified GCEs, respectively (Figure 5B). For the characterization of biocatalytic transformations on modified electrode surfaces, EIS is a commonly used efficient electrochemical technique. EIS gives information about the capacitance and load transfer resistance of the modified electrode’s surface. The charge transfer resistance (*R*ct) of K_3_[Fe(CN)_6_] was calculated using the diameters of the semicircles created in the Nyquist plots of the EIS spectra for the modified GC electrodes. When the electrode surface was modified, the load-transfer transition became more difficult, and the diameter of the semicircle increased. The *R*ct values belonging to bare GCE, GCE/PCL–CHIT/PAMAM_G1_–Mt, and GCE/PCL–CHIT/PAMAM_G1_–Mt/GluO*x* were increased step by step (Figure 5C).

### 3.3. PCL–CHIT/PAMAM_G1_–Mt/GluOx for MSG Detection

**Figure 6:**
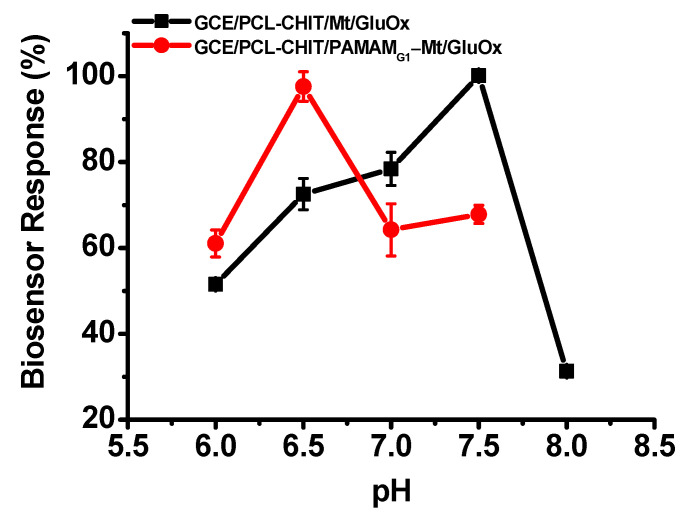
Optimum pH for the GCE/PCL–CHIT/Mt/GluO*x* and GCE/PCL–CHIT/PAMAM_G1_–Mt/GluO*x* biosensors (in 50 mM sodium phosphate buffer while stirring at room temperature; at −0.7 V; error bars show the standard deviations of three measurements).

The first optimization step of the working medium trials was the determination of optimum pH. The effects of pH on the biosensors’ responses were analyzed using PCL–CHIT/Mt/GluO*x* and PCL–CHIT/PAMAM_G1_–Mt/GluO*x* biosensors in 50 mM sodium phosphate buffer (from pH 6.0 to 8.0) while adding 0.25 mM MSG into the working cell as a substrate. Thus, the effect of PAMAM_G1_ on the nanofiber structure was tested to compare two developed biosensors. The amperometric biosensor’s responses were observed in µA and calculated relatively. As a result of the measurements, the optimum pH values of the PCL–CHIT/Mt/GluO*x* and PCL–CHIT/PAMAM_G1_–Mt/GluO*x* biosensors were 7.5 and 6.5 in a sodium phosphate buffer, respectively. As shown in Figure 6, the pH value shifted from an alkaline to an acidic region. The presence of amino groups in the PAMAM_G1_ dendrimer could be considered why the optimum pH of the PCL–CHIT/PAMAM_G1_–Mt/GluO*x* biosensor was more acidic [29,40].

**Figure 7:**
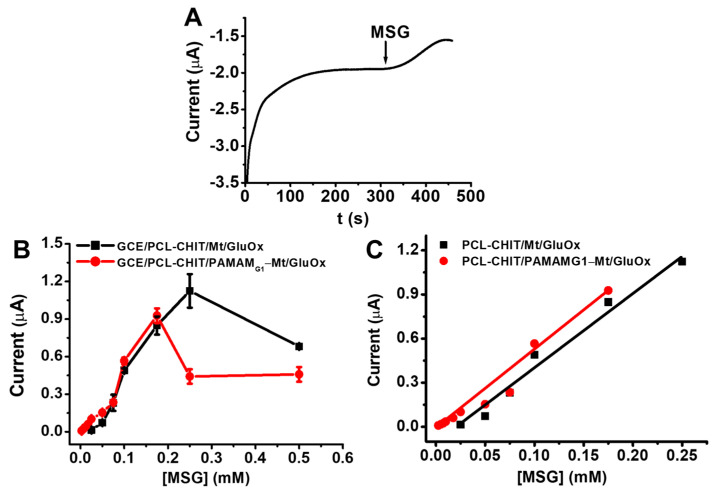
(**A**) Current change with the addition of MSG into the working buffer. (**B**) Responses of the GCE/PCL–CHIT/Mt/GluO*x* and GCE/PCL–CHIT/PAMAM_G1_–Mt/GluO*x* biosensors to various MSG concentrations. (**C**) Calibration graph of both biosensor systems (in 50 mM sodium phosphate buffer, pH 7.5 for GCE/PCL–CHIT/Mt/GluO*x* and pH 6.5 for GCE/PCL–CHIT/PAMAM_G1_–Mt/GluO*x*, at −0.7 V; error bars show the SDs of three measurements).

As a result of the enzymatic catalysis reaction of GluO*x*, the current was changed over time. The current variations after the addition of MSG are displayed in Figure 7A. Both developed biosensor system responses decreased at 0.5 mM of MSG (Figure 7B). The linear ranges were determined to be from 0.025 to 0.25 mM of MSG as a substrate using the equation *y* = 4.953*x* − 0.080 (*R*^2^ = 0.974) for the PCL–CHIT/Mt/GluO*x* biosensor and from 0.0025 to 0.175 mM MSG using the equation *y* = 5.423*x* − 0.023 (*R*^2^ = 0.985) for the PCL–CHIT/PAMAM_G1_–Mt/GluO*x* biosensor (with a limit of detection of 7.019 µM for PCL–CHIT/Mt/GluO*x* and 1.045 µM for GCE/PCL–CHIT/PAMAM_G1_–Mt/GluO*x* [n:8]) (Figure 7C). The sensitivities were 4.953 and 5.423 µA mM^−1^ for PCL–CHIT/Mt/GluO*x*, and PCL–CHIT/PAMAMG_1_–Mt/GluO*x*, respectively. This way, lower MSG concentrations could be detected using this biosensor system developed by modifying Mt with PAMAM_G1_. Due to PAMAM_G1_-modified Mt, the immobilization process was more successful at increasing the distances between the layers of clays in the PCL–CHIT/PAMAM_G1_–Mt/GluOx biosensor. Table 1 compares the performances of GluO*x*-based electrochemical biosensors in the literature.

One of the most important characterization studies of biosensors is repeatability trials. For an ideal biosensor system, almost identical results are expected to be obtained in consecutive measurements under the same conditions with the same electrode. The lower standard deviation (SD) and coefficient of variation (cv) indicate the reproducible biosensor system. For this purpose, five consecutive measurements were recorded with 0.0175 mM MSG using the developed biosensor system. According to these measured values, SD and CV were calculated as ±0.0016 and 5.585%, respectively. Herein, immobilizing the GluO*x* enzyme onto the nanofiber-coated electrode surface with glutaraldehyde as a cross-covalent binding agent provides high repeatability. Furthermore, for the operational stability determination of the PCL–CHIT/PAMAM_G1_–Mt/GluO*x* biosensor, amperometric measurements were recorded every 0.5 h for 6 h using 0.0175 mM MSG as a substrate under the same conditions. No significant decrease in activity was observed for the first 5 h, though the biosensor’s activity decreased by 63.415% at the end of 6 h.

**Figure 8:**
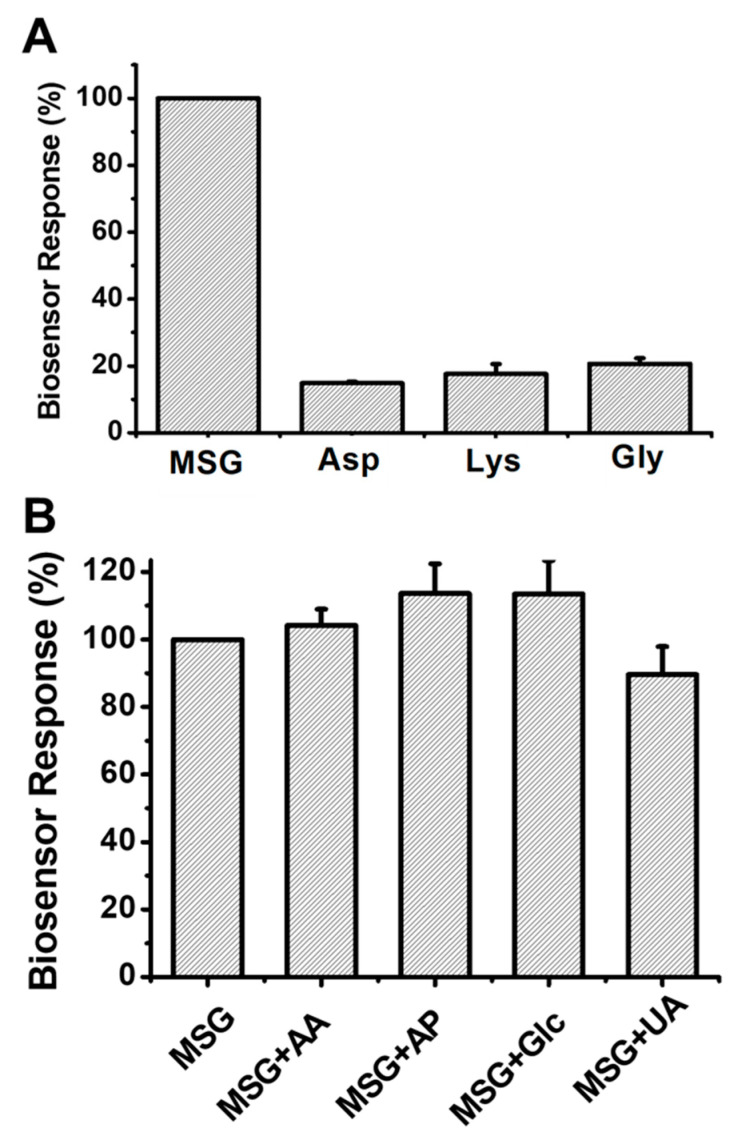
(**A**) Substrate specificity and (**B**) interference effects of some organic compounds on the response of the GCE/PCL–CHIT/PAMAM_G1_–Mt/GluO*x* biosensor (Asp: aspartic acid, Lys: lysine, Gly: glycine, AA: ascorbic acid; AP: 3-acetamidophenol; Glc: glucose, UA: uric acid; in 50 mM, pH 6.5 sodium phosphate buffer, at −0.7 V; error bars show the SDs of three measurements).

To determine the substrate specificity of PCL–CHIT/PAMAM_G1_–Mt/GluO*x*, measurements were taken using aspartic acid, lysine, and glycine as substrates, and the results were relatively comparable (Figure 8A). To examine whether the PCL–CHIT/PAMAM_G1_–Mt/GluO*x* biosensor was open to interference, measurements were performed by adding ascorbic acid, 3-acetamidophenol, glucose, and uric acid at the same concentrations to 0.025 mM MSG in the working buffer. The biosensor response to MSG was assumed to be 100%, and the responses to other components were compared relatively [59]. The results show no significant interference effect of the other compounds (Figure 8B).

### 3.4. PCL–CHIT/PAMAM_G1_–Mt/GluOx for MSG Detection in Real Samples

To test the applicability of the PCL–CHIT/PAMAM_G1_–Mt/GluO*x* biosensor to real samples, MSG determination was performed in tomato soup. Firstly, 22 g powdered soup was dissolved in 170 mL water and centrifuged for 15 min at 7000 rpm. Then, this supernatant was diluted 250 times with water, and a few drops of concentrated HCl were added to the solution. Amperometric measurements were taken using the prepared MSG added soup (by standard addition method) solution, and the biosensor’s response followed. According to the equation *y* = 5.423*x* − 0.023 [*R*^2^ = 0.985] for the PCL–CHIT/PAMAM_G1_–Mt/GluO*x* biosensor, the concentrations of standard MSG solution and MSG-added soup were found to be 0.030 ± 0.0025 (mM ± SD) and 0.029 ± 0.006 (mM ± SD), respectively. In line with these findings, recovery was calculated as 103.125%, indicating the successful application of the developed biosensor system to real samples.

## 4. Conclusions

PCL–CHIT/PAMAM_G1_–Mt/GluO*x* enzymatic biosensor was prepared and tested for sensitive, specific, and fast detection of MSG in real samples. First, Mt was intercalated with PAMAM, and the obtained PAMAM_G1_–Mt was successfully incorporated with the PCL–CHIT structure. PCL–CHIT/PAMAM_G1_–Mt was an alternative matrix for the covalent conjugation of GluO*x* to prepare the MSG biosensor. The usability of biosensors in the food industry was studied. The PCL–CHIT/PAMAM_G1_–Mt/GluO*x* has good features, which can be integrated with point-of-care sensor technologies.

## Figures and Tables

**Table 1:** A comparison of the performances of GluO*x*-based electrochemical biosensors in the literature.

Material	Detection Mode	Linear Range	LOD	Samples	Ref.
CuO with MWCNTs	CV	20–200 µM	17.5 µM	Whole blood and urine	[52]
GLDH/Chit-AA-CDs/SPCE	CV	11–125 µM	3.3 µM	Blood serum and barbecue flavored corn snack samles	[53]
ChBD-GluOX/PB/SPC	CV	25 µmol/L to 300 µmol/L	9.0 µmol/L	Fermentation broth samples	[54]
GluOx/PMPD/Pt modified GRE	CV	2.0–550 μM	0.536 μM	Cucumber juice and fruit	[55]
PtNP decorated MXene-Ti_3_C_2_T_x_	AMP	10–110 μmol/L	0.45 μmol/L	Vegetable soup, soy sauce, stock cube, and mushroom seasoning	[1]
Au@MoS_2_/CS	CV, DPV and EIS	0.05–200 µM	0.03 μM	Food	[13]
PPy/GluOx	AMP	5.0 µM–1.0 mM	1.8 μM	Stock cubes, ketchup and Parmigiano Reggiano chees	[56]
AuNPs/GO/CS	CV and DPV	0.2–1.4 mM	0.023 mM	Beef	[57]
CS-AuNPs	CV, DPV and EIS	100 pM to 1 μM	-	Freshly prepared tomato sauce	[14]
PANI-TiO_2_	CV and DPV	1 nM to 500 µM and 1 µM to 250 µM	37 mA/nM	Tomato sauce	[58]
PCL:CHIT/Mt	AMP	25 µM to 0.25 mM	7.019 µM	-	This study
PCL:CHIT/PAMAMG_1_-Mt	AMP	2.5 µM to 0.175 mM	1.045 µM	Tomato soup	This study

CuO: copper oxide; MWCNTs: multiwall carbon nanotubes; CV: cyclic voltammetry; GLDH/Chit-AA-CDs/SPCEs: L-glutamic dehydrogenase/chitosan carbon nanodots modified with azure A/screen printed carbon electrodes; ChBD-GluOx/PB/SPC: chitin-binding domain–glutamate oxidase/screen-printed Prussian blue nanocube microchip with the biopolymer chitosan; AMP: amperometric; GluOx/PMPD/Pt modified GRE: glutamate oxidase/poly (*m*-phenylenediamine) film/platinum modified graphite rot electrode; PtNP decorated MXene-Ti_3_C_2_T_x_: Pt nanoparticles (PtNP) decorated with two-dimensional nanomaterial MXene-Ti_3_C_2_T_x_; Au@MoS_2_/CS: molybdenum disulfide/chitosan; DPV: differential pulse voltammetry; EIS: electrochemical impedance spectroscopy; PPy: polypyrrole; AuNPs: gold nanoparticles; GO: graphene oxide; CS: chitosan; PANI-TiO_2_: polyaniline titanium oxide.

## Data Availability

Not applicable.

## References

[1] Liu J., Fan Y., Chen G., Liu Y. (2021). Highly Sensitive Glutamate Biosensor Based on Platinum Nanoparticles Decorated MXene-Ti3C2Tx for l-Glutamate Determination in Foodstuffs. LWT.

[2] Greenamyre J.T., Penney J.B., Young A.B., D’Amato C.J., Hicks S.P., Shoulson I. (1985). Alterations in L-Glutamate Binding in Alzheimer’s and Huntington’s Diseases. Science.

[3] Pépin J., Francelle L., Carrillo-de Sauvage M.-A., de Longprez L., Gipchtein P., Cambon K., Valette J., Brouillet E., Flament J. (2016). In Vivo Imaging of Brain Glutamate Defects in a Knock-in Mouse Model of Huntington’s Disease. NeuroImage.

[4] Thongsepee N., Martviset P., Chantree P., Sornchuer P., Sangpairoj K., Prathaphan P., Ruangtong J., Hiranyachattada S. (2022). Daily Con-sumption of Monosodium Glutamate Pronounced Hypertension and Altered Renal Excretory Function in Normotensive and Hypertensive Rats. Heliyon.

[5] Baad-Hansen L., Cairns B., Ernberg M., Svensson P. (2010). Effect of Systemic Monosodium Glutamate (MSG) on Headache and Pericranial Muscle Sensitivity. Cephalalgia.

[6] Zanfirescu A., Ungurianu A., Tsatsakis A.M., Nițulescu G.M., Kouretas D., Veskoukis A., Tsoukalas D., Engin A.B., Aschner M., Margină D. (2019). A Review of the Alleged Health Hazards of Monosodium Glutamate. Compr. Rev. Food Sci. Food Saf..

[7] Kurkinen M., Pavlovic Z.M. (2022). Astrocyte Glutamate Transporter EAAT2 in Alzheimer Dementia. Glutamate and Neuropsychiatric Disorders: Current and Emerging Treatments.

[8] Scoggin J.L., Tan C., Nguyen N.H., Kansakar U., Madadi M., Siddiqui S., Arumugam P.U., DeCoster M.A., Murray T.A. (2019). An Enzyme-Based Electrochemical Biosensor Probe with Sensitivity to Detect Astrocytic versus Glioma Uptake of Glutamate in Real Time in Vitro. Biosens. Bioelectron..

[9] Chung Y., Yu D., Kwak H.S., Park S.S., Shin E.C., Lee Y. (2022). Effect of Monosodium Glutamate on Salt and Sugar Content Reduction in Cooked Foods for the Sensory Characteristics andConsumer Acceptability. Foods.

[10] Abdallah C.G., Jiang L., De Feyter H.M., Fasula M., Krystal J.H., Rothman D.L., Mason G.F., Sanacora G. (2014). Glutamate Metabolism in Major Depressive Disorder. AJP.

[11] Okon S.L., Ronkainen N.J., Okon S.L., Ronkainen N.J. (2017). Enzyme-Based Electrochemical Glutamate Biosensors.

[12] Nanda P.K., Bhattacharya D., Das J.K., Bandyopadhyay S., Ekhlas D., Lorenzo J.M., Dandapat P., Alessandroni L., Das A.K., Ga-gaoua M. (2022). Emerging Role of Biosensors and Chemical Indicators to Monitor the Quality and Safety of Meat and Meat Products. Chemosensors.

[13] Devi R., Gogoi S., Barua S., Sankar Dutta H., Bordoloi M., Khan R. (2019). Electrochemical Detection of Monosodium Glutamate in Foodstuffs Based on Au@MoS2/Chitosan Modified Glassy Carbon Electrode. Food Chem..

[14] Sharma D., Devi R., Jaiswal J., Dutta H.S., Khan R., Dhayal M. (2022). A Highly Sensitive Immunosensor Based on In Situ Reduced Gold-Chitosan Nanocomposite for Detection of Monosodium L-Glutamate. J. Biosyst. Eng..

[15] Li P., Jia J., Geng Z., Pang S., Wang R., Bilal M., Bian H., Cui J., Jia S. (2023). A Dual Enzyme-Phosphate Hybrid Nanoflower for Glutamate Detection. Particuology.

[16] Santos F.D.S., Silva L.V.d., Campos P.V.S., Strunkis C.d.M., Ribeiro C.M.G., Salles M.O. (2022). Review—Recent Advances of Electro-chemical Techniques in Food, Energy, Environment, and Forensic Applications. ECS Sens. Plus.

[17] Demirkol D.O., Yildiz H.B., Sayın S., Yilmaz M. (2014). Enzyme Immobilization in Biosensor Constructions: Self-Assembled Monolayers of Calixarenes Containing Thiols. RSC Adv..

[18] Oner A., Tufek E., Yezer I., Birol A., Demir M., Er S., Demirkol D.O. (2021). High Generation Dendrimer Decorated Poly-Ɛ-Caprolactone/Polyacrylic Acid Electrospun Nanofibers for the Design of a Bioelectrochemical Sensing Surface. React. Funct. Polym..

[19] Serafín V., Valverde A., Martínez-García G., Martínez-Periñán E., Comba F., Garranzo-Asensio M., Barderas R., Yáñez-Sedeño P., Campuzano S., Pingarrón J.M. (2019). Graphene Quantum Dots-Functionalized Multi-Walled Carbon Nanotubes as Nanocarriers in Electrochemi-cal Immunosensing. Determination of IL-13 Receptor A2 in Colorectal Cells and Tumor Tissues with Different Metastatic Potential. Sens. Actuators Chem..

[20] Kırgöz Ü.A., Odacı D., Timur S., Merkoçi A., Pazarlıoğlu N., Telefoncu A., Alegret S. (2006). Graphite Epoxy Composite Electrodes Modified with Bacterial Cells. Bioelectrochemistry.

[21] Çakar İ., Özdokur K.V., Demir B., Yavuz E., Demirkol D.O., Koçak S., Timur S., Ertaş F.N. (2013). Molybdenum Oxide/Platinum Modified Glassy Carbon Electrode: A Novel Electrocatalytic Platform for the Monitoring of Electrochemical Reduction of Oxygen and Its Biosensing Applications. Sens. Actuators Chem..

[22] Bongartz R., Ag D., Seleci M., Walter J.-G., Yalcinkaya E.E., Demirkol D.O., Stahl F., Timur S., Scheper T. (2012). Folic Acid-Modified Clay: Targeted Surface Design for Cell Culture Applications. J. Mater. Chem..

[23] Sonmez B., Sayin S., Yalcinkaya E.E., Seleci D.A., Yildiz H.B., Demirkol D.O., Timur S. (2014). Calixarene Modified Montmorillonite: A Novel Design for Biosensing Applications. RSC Adv..

[24] Yilmaz Y.Y., Yalcinkaya E.E., Demirkol D.O., Timur S. (2020). 4-Aminothiophenol-Intercalated Montmorillonite: Organic-Inorganic Hybrid Material as an Immobilization Support for Biosensors. Sens. Actuators Chem..

[25] Demir B., Seleci M., Ag D., Cevik S., Yalcinkaya E.E., Demirkol D.O., Anik U., Timur S. (2013). Amine Intercalated Clay Surfaces for Mi-crobial Cell Immobilization and Biosensing Applications. RSC Adv..

[26] Songurtekin D., Yalcinkaya E.E., Ag D., Seleci M., Demirkol D.O., Timur S. (2013). Histidine modified montmorillonite: Laccase immobilization and application to flow injection analysis of phenols. Appl. Clay Sci..

[27] Seleci M., Ag D., Yalcinkaya E.E., Demirkol D.O., Guler C., Timur S. (2012). Amine-intercalated montmorillonite matrices for enzyme immobilization and biosensing applications. RSC Adv..

[28] Unal B., Yalcinkaya E.E., Gumustas S., Sonmez B., Ozkan M., Balcan M., Demirkol D.O., Timur S. (2017). Polyglycolide–montmorillonite as a novel nanocomposite platform for biosensing applications. New J. Chem..

[29] Unal B., Yalcinkaya E.E., Demirkol D.O., Timur S. (2018). An Electrospun Nanofiber Matrix Based on Organo-Clay for Biosensors: PVA/PAMAM-Montmorillonite. Appl. Surf. Sci..

[30] Kirbay F.O., Yalcinkaya E.E., Atik G., Evren G., Unal B., Demirkol D.O., Timur S. (2018). Biofunctionalization of PAMAM-Montmorillonite Decorated Poly (Ɛ-Caprolactone)-Chitosan Electrospun Nanofibers for Cell Adhesion and Electrochemical Cytosensing. Biosens. Bioelectron..

[31] Damar K., Odaci Demirkol D. (2011). Modified Gold Surfaces by Poly(Amidoamine) Dendrimers and Fructose Dehydrogenase for Mediated Fructose Sensing. Talanta.

[32] Hatamluyi B., Es’haghi Z. (2018). Quantitative Biodetection of Anticancer Drug Rituxan with DNA Biosensor Modified PAMAM Den-drimer/Reduced Graphene Oxide Nanocomposite. Electroanalysis.

[33] Malvano F., Pilloton R., Rubino A., Albanese D. (2022). Rapid Detection of Deoxynivalenol in Dry Pasta Using a Label-Free Immunosensor. Biosensors.

[34] Wang X., Shi W., Wang Y., Cheng D., Liu J., Xu S., Liu W., Dong B., Sun J. (2022). Intrinsic Blue Fluorescence of 2.0G PAMAM-DCM Polymer Dots and Its Applications for Fe^3+^ Sensing. Sensors.

[35] Idris A.O., Akanji S.P., Orimolade B.O., Olorundare F.O.G., Azizi S., Mamba B., Maaza M. (2023). Using Nanomaterials as Excellent Immobilisation Layer for Biosensor Design. Biosensors.

[36] Du Y., Zhang X., Liu P., Yu D.-G., Ge R. (2022). Electrospun Nanofiber-Based Glucose Sensors for Glucose Detection. Front. Chem..

[37] Andrady A.L. (2022). Nanofiber-Based Chemical Sensors. Applications of Polymer Nanofibers.

[38] Kim D.-H., Bae J., Lee J., Ahn J., Hwang W.-T., Ko J., Kim I.-D. (2022). Porous Nanofiber Membrane: Rational Platform for Highly Sensitive Thermochromic Sensor. Adv. Funct. Mater..

[39] Chokkiah B., Eswaran M., Wabaidur S.M., Alothman Z.A., Lee S.C., Dhanusuraman R. (2022). An Efficient Amperometric Sensor for Chloride Ion Detection through Electroactive E-Spun PVA-PANi-g-C3N4 Nanofiber. J. Mater. Sci. Mater. Electron..

[40] Yezer I., Demirkol D.O. (2020). Cellulose Acetate–Chitosan Based Electrospun Nanofibers for Bio-Functionalized Surface Design in Biosensing. Cellulose.

[41] Du J., Tan E., Kim H.J., Zhang A., Bhattacharya R., Yarema K.J. (2014). Comparative Evaluation of Chitosan, Cellulose Acetate, and Polyether-sulfone Nanofiber Scaffolds for Neural Differentiation. Carbohydr. Polym..

[42] Shabanloo R., Akbari S., Mirsalehi M. (2022). Hybrid electrospun scaffolds based on polylactic acid/PAMAM dendrimer/gemini surfactant for enhancement of synergistic antibacterial ability for biomedical application. Biomed. Mater..

[43] Priya, Gogate P.R. (2022). Ultrasound-Assisted Intensification of β-Glucosidase Enzyme Activity in Free and Immobilized Forms. Ind. Eng. Chem. Res..

[44] Tangaraj V., Janot J.-M., Jaber M., Bechelany M., Balme S. (2017). Adsorption and Photophysical Properties of Fluorescent Dyes over Montmo-rillonite and Saponite Modified by Surfactant. Chemosphere.

[45] Song C., Yu H., Zhang M., Yang Y., Zhang G. (2013). Physicochemical Properties and Antioxidant Activity of Chitosan from the Blowfly *Chrysomya megacephala* Larvae. Int. J. Biol. Macromol..

[46] Costa M.S.S.P., Costa L.S., Cordeiro S.L., Almeida-Lima J., Dantas-Santos N., Magalhães K.D., Sabry D.A., Albuquerque I.R.L., Pe-reira M.R., Leite E.L. (2012). Evaluating the Possible Anticoagulant and Antioxidant Effects of Sulfated Polysaccharides from the Tropical Green Alga *Caulerpa Cupressoides* Var. Flabellata. J. Appl. Phycol..

[47] Jeon H.J., Kim J.S., Kim T.G., Kim J.H., Yu W.-R., Youk J.H. (2008). Preparation of Poly(ɛ-Caprolactone)-Based Polyurethane Nanofibers Containing Silver Nanoparticles. Appl. Surf. Sci..

[48] Quoc P.L., Solovieva A.Y., Uspenskaya M.V., Olekhnovich R.O., Sitnikova V.E., Strelnikova I.E., Kunakova A.M. (2021). High-Porosity Polymer Composite for Removing Oil Spills in Cold Regions. ACS Omega.

[49] Yu Y., Zhang B., Wang Y., Qi G., Tian F., Yang J., Wang S. (2016). Co-continuous structural electrolytes based on ionic liquid, epoxy resin and organoclay: Effects of organoclay content. Mater. Des..

[50] Zhang S., Zhao G., Wang J., Xie C., Liang W., Chen K., Wen Y., Li X. (2021). Organic Solvent-Free Preparation of Chitosan Nanofibers with High Specific Surface Charge and Their Application in Biomaterials. ACS Appl. Mater. Interfaces.

[51] Yotsuji K., Tachi Y., Sakuma H., Kawamura K. (2021). Effect of interlayer cations on montmorillonite swelling: Comparison between molecular dynamic simulations and experiments. Appl. Clay Sci..

[52] Ali M.Y., Knight D., Howlader M.M.R. (2023). Nonenzymatic Electrochemical Glutamate Sensor Using Copper Oxide Nanomaterials and Multi-wall Carbon Nanotubes. Biosensors.

[53] Martínez-Periñán E., Domínguez-Saldaña A., Villa-Manso A.M., Gutiérrez-Sánchez C., Revenga-Parra M., Mateo-Martí E., Pariente F., Lorenzo E. (2023). Azure A Embedded in Carbon Dots as NADH Electrocatalyst: Development of a Glutamate Electrochemical Biosensor. Sens. Actuators Chem..

[54] Yang L., Bai R., Xie B., Zhuang N., Lv Z., Chen M., Dong W., Zhou J., Jiang M. (2023). A Biosensor Based on Oriented Immobilization of an Engineered L-Glutamate Oxidase on a Screen-Printed Microchip for Detection of l-Glutamate in Fermentation Processes. Food Chem..

[55] Lin Y., Yang L., Ma Y., Ye J. (2023). Construction of Minitype Glutamate Sensor for in Vivo Monitoring of L-Glutamate in Plant. Microchem. J..

[56] Mentana A., Nardiello D., Palermo C., Centonze D. (2020). Accurate Glutamate Monitoring in Foodstuffs by a Sensitive and Interference-Free Glutamate Oxidase Based Disposable Amperometric Biosensor. Anal. Chim. Acta.

[57] Wang X., Duan J., Cai Y., Liu D., Li X., Dong Y., Hu F. (2020). A Modified Nanocomposite Biosensor for Quantitative L-Glutamate Detection in Beef. Meat Sci..

[58] Sharma D., Dutta H.S., Dhayal M. (2022). Langmuir-Blodgett Monolayer of Electrochemically Synthesized PANI-TiO2 Nanocomposites for MSG Biosensor. Appl. Surf. Sci. Adv..

[59] Shigemura N., Shirosaki S., Sanematsu K., Yoshida R., Ninomiya Y. (2009). Genetic and Molecular Basis of Individual Differences in Human Umami Taste Perception. PLoS ONE.

